# Histopathological and Immune Prognostic Factors in Colo-Rectal Liver Metastases

**DOI:** 10.3390/cancers13051075

**Published:** 2021-03-03

**Authors:** Alessandra Rigamonti, Friedrich Feuerhake, Matteo Donadon, Massimo Locati, Federica Marchesi

**Affiliations:** 1 Department of Immunology and Inflammation, IRCCS Humanitas Research Hospital, 20089 Rozzano, Italy; alessandra.rigamonti@humanitasresearch.it (A.R.); massimo.locati@humanitasresearch.it (M.L.); 2 Department of Biotechnology and Translational Medicine, University of Milan, 20129 Milan, Italy; 3 Institute of Pathology, Hannover Medical School, D-30625 Hannover, Germany; feuerhake.friedrich@mh-hannover.de; 4 Department of Hepatobiliary and General Surgery, IRCCS Humanitas Research Hospital, 20089 Rozzano, Italy; matteo.donadon@hunimed.eu; 5 Department of Biomedical Sciences, Humanitas University, 20090 Pieve Emanuele, Italy

**Keywords:** colo-rectal liver metastasis, growth patterns, tumor regression score, macrophages, prognosis

## Abstract

**Simple Summary:**

Clinical management of colo-rectal liver metastasis would benefit from a refined stratification of patients in prognostic groups, in order to identify the best therapeutic option. Efforts are ongoing in the definition of parameters associated with clinical behaviors, which could help classifying patients in clinically relevant groups. Here we aimed at discussing the recent advances in this field, and we introduced current and new promising candidates, such as morphological tumor features and immune components, which have been showing significant association with survival. Some of these parameters are slowly reaching the clinic and further efforts are ongoing in the attempt to combine them in multiparametric scores.

**Abstract:**

Prognostic studies are increasingly providing new tools to stratify colo-rectal liver metastasis patients into clinical subgroups, with remarkable implications in terms of clinical management and therapeutic choice. Here, the strengths and hurdles of current prognostic tools in colo-rectal liver metastasis are discussed. Alongside more classic histopathological parameters, which capture features related to the tumor component, such as tumor invasion, tumor growth pattern and regression score, we will discuss immune mediators, which are starting to be considered important features. Their objective quantification has shown significant results in prognostication studies, with most of the work focused on adaptive immune cells, namely T cells. As for macrophages, they are only starting to be appreciated and we will present recent advances in evaluation of macrophage morphological features. Deeper knowledge acquired by multiparametric analyses is rapidly uncovering the variety of immune players that should be assessed. The future projection is to implement deep-learning histopathological tools and to integrate histopathological and immune metrics in multiparametric scores, with the ultimate objective to achieve a deeper resolution of the tumor features and their relevance for colo-rectal liver metastasis.

## 1. Introduction

Based on the last update of the World Health Organization (WHO), colorectal cancer (CRC) represents the third most common cancer worldwide as well as the second cause of cancer mortality [1]. The liver is the primary site of metastasis, being the main filter of the venous drainage of the gastro-intestinal tract, and approximately 50% of CRC patients develop colorectal liver metastases (CLM). A subgroup of respectable patients benefits from surgical resection of metastatic nodules, reaching a five-year survival rate of 50–60% [2]. Eligibility of CLM patients for surgical resection has progressively increased due to improvements of neo-adjuvant regimens and more effective combinations of chemotherapy and target therapies (such as anti-VEGF and anti-EGFR), which have offered the possibility of hepatic resection to a significant proportion of patients with high tumor burden. Notwithstanding, a considerable fraction of patients, up to 70%, recurs after CLM resection and the identification of this high-risk population has become a challenging clinical need. In this scenario, the identification of variables associated with disease progression becomes of seminal importance to improve patient stratification and ameliorate clinical output. Traditional tumor-related factors, including TNM staging, anatomical location of the primary tumor and status of the resection margin, are informative tools to classify CLM patients according to their prognosis. However, clinical presentations of patients belonging to the same staging group can be quite diverse as well as varying degrees of responsiveness to therapy are observed. Collectively, there is tremendous effort in the identification and implementation of new prognostic markers.

In recent years the prognostication of CLM has changed, introducing new classifiers that could perform better in terms of patient stratification. Much inspiration has been taken from the parameters used in the primary CRC setting, however, while for primary CRC pathologists routinely report these parameters, a narrower description of tumor features is provided for CLM. Here we will review current histopathological parameters as well as immune mediators that are emerging as novel classifiers in CLM. Many of the studies done in this clinical setting reflect the progress made in the prognostication of colo-rectal cancer, in which the consensus immunoscore, i.e., the quantitative assessment of number of T and cytotoxic T cells in tumor regions, has proven a strong prognostic relevance [3,4,5]. Further studies are needed to implement their use in the clinical routine of CLM.

The aim of this review is to present the available data that are propelling the introduction of new factors in human CLM prognostication. Among the variety of biomarkers currently evaluated, we will focus our attention on those parameters that can be assessed on histological sections (histological parameters) and that have shown prognostic impact in available studies. This choice is explained by the possibility to integrate histopathological parameters, primarily related to tumor features, with immune parameters, which consider the number, type and localization of immune cells in cancer tissues [6]. Provided that measurable parameters are generated, these could be integrated into prognostic scores, potentially complementing the ones available.

## 2. Histopathological Parameters

Over the years, a fruitful integration among surgeons and pathologists has contributed to identify some critical parameters that can be reproducibly and quantitatively assessed in resected metastasis specimens and that have shown significant prognostic relevance [7,8,9]. These include macroscopic parameters, such as size and number of lesions, molecular classifiers, such as the presence of *RAS* and *BRAF* mutations, as well as histopathological variables, particularly the metastatic tumor invasion, the tumor histopathological growth pattern (HGP), the tumor regression grading (TRG) and the chemotherapy-associated liver injury (CALI). Discussion is still lively on which histopathological characteristic should be evaluated and included in the pathological report.

### 2.1. Metastatic Tumor Invasion

The spreading of tumor cells occurs via different routes, including via vascular and lymphatic vessels, bile ducts and along the nerves. The presence of cancer cells within or in close proximity to these areas can therefore be considered a potential prognostic histopathological feature. Colorectal cancer cells can potentially spread within or outside the organ using the same pathways and originate liver metastases [8,10].

Intrahepatic vascular invasion refers to the occurrence of tumor invasion in either hepatic or portal veins. It is common (although not obvious) to evaluate the potential of invasion of the portal vein or hepatic vein as two distinct elements. To date, the clinical relevance of intrahepatic vascular invasion in CLM patients is still controversial. Some studies have reported hepatic and portal vein invasion to have poor prognostic significance, either as independent histopathological feature or in combination with others, such as lymphatic invasion [11,12,13,14]. However, other studies have not identified any association between vascular invasion and prognosis of resected CLM patients [15]. Further analyses are therefore needed to clarify this matter.

A similar argument can be formulated with lymphatic invasion, i.e., the presence of neoplastic cells within the luminal structure lined by endothelial cells in the portal area. The correlation between this model of tumor spreading and prognosis, initially studied in intrahepatic cholangiocarcinoma, was first investigated in CLM patients by Sasaki et al., using standard H&E [16]. In this particular study, the lymphatic invasion resulted in significantly and adversely impacting the overall, disease-free and extrahepatic-disease-free survival. Since then, the majority of the subsequent analyses have shown similar results [13,14,15], while others have reported that lymphatic invasion alone does not constitute a predictor of a worse prognosis [17]. It has been speculated that these controversial observations depend on the method adopted to determine the lymphatic invasion, given that most of the studies reporting a significant impact on the outcome used immunohistochemistry rather than H&E. However, lymphatic invasion has been recently evaluated in a cohort of 229 CLM patients through the detection of podoplanine (a lymphatic endothelium marker) by the D2-40 antibody, and no significant association with prognosis was found [14].

The invasion of biliary ducts is another variable that has been considered as a possible means of stratifying patients according to their outcomes. When this parameter is investigated by immunohistochemistry, biliary duct epithelium is highlighted by targeting cytokeratin-7. Moreover, the visualization of tumor cells from the colon mucosa (positive for CDX2 and CK20) may help to differentiate between metastatic colon cancer, showing intrabiliary growth, and other cancers, such as primitive biliary neoplasias (always positive for CK7, like nontumoral bile ducts) or hepatocellular carcinoma (positive for glypican-3, GPC3) with bile duct invasion [16,18,19]. Bile duct involvement, due to CRC, has been described in various case reports and case study series (see review [20]). However, the utility of annotating this parameter in pathology reports is still under debate [8,19]. Indeed, distinct studies came up with opposite results: some authors have not found any correlation between biliary invasion and clinical prognosis [11,13,15,17], while others showed a better prognosis for patients with invasion of bile ducts [18]. These discrepancies may be due to the different prognostic values associated with macroscopic or microscopic bile duct involvement. One critical point is related to classification, wherein analysis of the results is often hindered by a chaotic multiplicity of the terminology describing the phenomena. Indeed, terms like “liver metastasis”, “intrabiliary extension”, “biliary invasion of colorectal metastasis”, “intra bile duct metastases from CRC”, “intrabiliary metastases in CRC”, are often used indiscriminately to define different situations: either macroscopic/microscopic bile duct involvement, with/without and with important/marginal lesions in the liver parenchyma. Harmonization of histopathological reports may contribute to better classification and disclose important biomarkers with clinical relevance.

Perineural invasion is an active phenomenon though which tumor cells migrate along axons, supported by the favorable microenvironment orchestrated by neoplastic cells, nerves and the immune system [21]. This feature, generally associated with poor clinical outcome, is well studied in such cancers where perineural invasion is a common characteristic, such as in pancreatic cancer [22,23]. Instead, the analysis of this parameter, conducted by H&E or S100 immunohistochemistry, is poorly investigated in resected CLMs. Only few authors addressed this topic and reported opposite results: some of them found a significant relationship between perineural invasion and worst prognosis [11,24], while some studies haven’t recognized perineural invasion as prognostic factor [13].

Overall, histopathological features of CLM represent important characteristics with reasonable impact on the course of disease, however, to date, there is a remarkable lack of consensus on their prognostic value and further analyses are needed to clarify this matter. Recently, de Oliveira et al. performed a systematic review of clinical observational studies and a meta-analysis aimed at identifying the impact of histopathological factors on the prognosis of resected CLM patients [9]. The analysis evaluated 33 studies inclusive of more than 4000 patients and took into account those parameters for which there was available data concerning the prognostic impact for overall and disease-free survival (i.e., low degree of differentiation, invasion of lymphatic and blood vessels, nerves and biliary ducts, presence of tumor budding, satellite nodules and tumor borders). Some parameters (such as tumor differentiation and lymphatic invasion) showed only tendencies and did not reach significant association, possibly due to the small number of patients included, while portal vein and perineural invasion and the presence of satellite nodules showed a significant association with worse outcome. Collectively, given these recent findings, the importance of portal vein for tumor spreading and the role of vascular invasion as a prognostic classifier in many gastrointestinal tumors (including CRC) [8], we agree with previous reports that the presence/absence of portal and hepatic vein invasion should be documented in the CLM pathology report, despite an immediate utility of these features in the daily clinical practice is far from being achieved.

### 2.2. Histopathological Growth Patterns (HGP)

Colo-rectal metastases show different manners to growth in the liver and to interact with the organ, as appears from the morphological variations that characterize the interface between the tumor and the surrounding normal tissue. The identification of distinct histopathological growth patterns (HGPs) has given the possibility to test this parameter as possible prognostic factor, revealing promising results in several studies.

**Figure 1:**
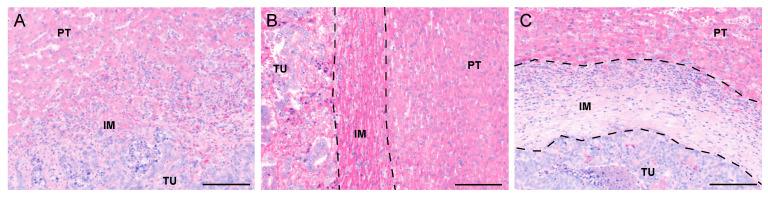
Representative images of the three major histopathological growth patterns (HGPs) in colorectal liver metastases identified on H&E-stained tissue sections: (**A**) replacement HGP, characterized by tumor cells infiltrating the surrounding liver parenchyma (IM region). (**B**) pushing HGP, recognized by the liver tissue compressed and pushed away by the tumor (IM region between the two dotted lines) and (**C**) desmoplastic HGP, identified by a rim of fibrotic tissue that encapsulates the metastasis (IM region between the two dotted lines). Scale bar: 50 μm. PT: peritumor, IM: invasive margin, TU: tumor regions.

Two systems are primarily used to classify the HGPs. To date, the most accepted system, according to the international consensus guidelines [25], identifies three main different HGPs: the “replacement”, the “pushing” and the “desmoplastic” patterns. Examples of these three tumor borders are shown in Figure 1. In the replacement pattern, tumor cells are in continuity with the liver parenchyma and replace the hepatocytes in their proximity; the pushing HGP presents cancer cell plates compressing the surrounding liver tissue without mimicking the architecture of the healthy organ; the desmoplastic patter is characterized by a rim of fibrous tissue that isolates the metastases from the liver parenchyma. Two other rare patterns have been described: the sinusoidal HGP, identified by tumor cells that grow within sinusoids and in the peri-sinusoidal spaces, and the portal HGP, defined by the presence of cancer cells growing in the connective tissue space of liver capsule, liver septa or portal tract [25]. The second classification transposes the criteria used in CRCs [26] to evaluate liver metastases, and distinguishes between “infiltrative” tumor border, characterized by the spreading of the metastasis through the surrounding liver tissue, and the “expansive” tumor border, identified in tumors having well-defined edges that push the adjacent healthy tissue. It is common that, in studies adopting this last classification system, the presence/absence of tumor pseudocapsule is considered as a distinct prognostic feature. In an attempt to harmonize, the infiltrative tumor border is easily matching with the replacement HGP, while the expansive and encapsuled tumor borders are more similar to the pushing HGP observed in CLM. Once the terminology has been conformed, it is possible to claim that, in the vast majority of studies, a significantly worse prognosis was reported in patients with the replacement HGP or the infrequent pushing HGP of metastases [25,27,28]. Conversely, the desmoplastic HGP has been associated with significantly better outcome [9,25,28,29].

The existence of multiple metastatic growth patterns reflects different interactions that the tumor can establish with the host, and vice versa. This concept partially explains the distinct outcomes observed in CLM patients according to their metastatic HGPs. Thus, the desmoplastic and pushing tumor borders are characterized by a large number of immature blood vessels and proliferating endothelial cells, reflecting the intense angiogenesis occurring in the metastases for the construction of its supporting stroma. Moreover, elevated lymphocyte infiltration in the tumor–liver interface is observed in this type of cancers. On the contrary, tumors with replacement growth pattern co-opt the blood vessels and the connective tissue of the surrounding healthy liver for their expansion and have a general non-inflamed status [30].

### 2.3. Tumor Regression Grade (TRG)

Partial hepatectomy to resect liver metastases represents a curative therapeutic approach in 20% of patients with CLM [31], but unfortunately only the minority of subjects is eligible for surgical resection. Hence there is an urgent need to increase the patient population that benefits from a surgical procedure. In recent years, neoadjuvant chemo-immunotherapy has proved to be an effective strategy in reducing the tumor burden, improving five-year overall survival in high-risk resectable liver metastases and increasing the chances for patients with primarily unresectable metastasis to be re-considered for hepatectomy after successful tumor reduction [32]. Oxaliplatin- and irinotecan-based chemotherapy with either the antivascular endothelial growth factor (VEGF) bevacizumab or, for KRAS wild-type tumors, the anti-epidermal growth factor receptor (EGFR) cetuximab and panitumumab, resulted in R0 resection in 27–38% of patients who initially would not have been considered for surgery [33]. Overall, tumor response to chemotherapy is one of the strongest prognostic factors after surgery [34], therefore, novel tools to assess the impact of preoperative chemotherapy are particularly relevant.

Macroscopical evaluation of tumor response to neoadjuvant treatments before surgery, using computed tomography or magnetic resonance, is thus crucial for the optimization of CLM management; however, microscopical evaluation of resected metastases after partial hepatectomy is also essential. Indeed, pathological response has been shown to be predictive of favorable prognosis [35,36]. Different histopathological methods have been developed to estimate tumor response to pre-operative therapies, including distinction between presence or absence of viable cancer cells (using H&E or, in case of doubts, immunostaining of CK20 and CK7 for a better visualization of colorectal tumor cells and dystrophic biliary structures, respectively) [36]; assessment of the percentage of viable tumor in relation to the total tumor area [35,37] and the evaluation of tumor thickness at the tumor-normal interface [38]. The tumor regression grade (TRG) score, initially proposed by Rubbia-Brandt et al. [39], is another method commonly used to evaluate chemo-immunotherapy tumor response. It is based on the assessment of the proportion between cancer cells and fibrosis in a scale of one to five, from TRG1, corresponding to complete replacement of the residual tumor by extensive fibrosis, to TRG5, representing cases of persistence of tumor cells without fibrosis.

Increase of T cell infiltration in resected CLM previously treated with chemotherapy has been reported [40,41]. A recent study has shown that the T cell density in the intratumor region is significantly increased in patients resected within a short-interval from neoadjuvant chemotherapy completion (evaluated by computer tomography or magnetic resonance images) compared to cases of long-interval between the preoperative treatment and the hepatectomy [42]. Moreover, density of tumor infiltrating lymphocytes is correlated with superior recurrence-free survival in patients with TRG scores of one or two [43]. These findings suggest that a better understanding of the strong relation among the tumor response to neoadjuvant treatments, the immune response to the tumor and the patient outcome would refine patient stratification and improve outcome prediction.

### 2.4. Chemotherapy-Associated Liver Injury (CALI)

Preoperative chemotherapy plays a critical role in the improvement of the management of CLM patients. Nonetheless, it presents some shortcomings, the most important being the development of chemotherapy-associated liver injuries (CALI), which include sinusoidal obstruction syndrome (SOS), nodular regenerative hyperplasia (NRH) and steatohepatitis. SOS can be considered a consequence of endothelial damage related to preoperative treatment. NRH would be the advanced stage, whereby nodular modification of the liver parenchyma is recorded [34,44]. In addition, different CALI are observed following different chemotherapy regimens and their occurrence increases with the duration of chemotherapy. Steatohepatatis needs to be carefully considered because it could be nonspecifically related to treatment rather to dysmetabolic conditions. The impact of CALI on postoperative outcome in patients undergoing partial hepatectomy for colorectal liver metastases remains controversial. A systematic review reported an increase in postoperative major morbidities in patients with severe sinusoidal dilatation and steatohepatitis [44]. In a recent study, Baldin et al. analyzed the liver injury associated to chemotherapy in a cohort of 187 CLM patients and recorded the presence of CALI in 68.9%, with SOS as the most frequent type (36.4%) [2].

## 3. Tissue Immune Parameters

The density and types of immune cells infiltrating cancer tissues, commonly referred to as the immune contexture [6,45], has been object of intense studies, which have been particularly relevant in the clinical setting of colo-rectal cancer and liver metastasis [3,4,5]. In terms of clinical applicability, these studies are projected to reach two main objectives, closely inter-related, though primarily fulfilled by opposite experimental approaches. The first objective is a largely observer-independent, high-resolution definition of the multiple immune cells populating the tumor tissue. The primary approach towards this goal includes multi-dimensional strategies, such as multiplexed morphological methods, including the new imaging mass spectrometry and laser-capture microdissection, or “-omics” technologies, like single cell RNA sequencing. A prime example of the power of such tools applied to the study of the tumor immune microenvironment in human liver malignances is offered by the recent work by Massalha et al. [46]. In this study, the authors analyzed tissue samples from patients who underwent hepatic resection, including three CLM patients, and reconstructed a complete zonation cell atlas of both the malignant and nonmalignant liver, providing a comprehensive analysis of the mechanisms of cross-talk between the tumor microenvironment and carcinomas, and of the tissue spatial gene expression profile. The second objective is to narrow down to specific biomarkers to be used in daily routine, which is ultimately going to restrict the analysis to a limited number of variables. A prominent example for this approach is the “Immunoscore”, a widely harmonized evaluation of the adaptive immune response to tumors, specifically T-cell and cytotoxic T-cell counts, representing a broadly accepted output of various studies in this field [4,47]. As for the innate immune response to malignancies, particularly myeloid cells including macrophages, a similar goal has not yet been achieved [48].

### 3.1. The Prognostic Relevance of T Cells in CLM

The integration of quantitative information related to the density, type and localization of T cells in colo-rectal cancer has been the strength of the Immunoscore. The universe of adaptive immune cells, namely T cells, and their activation states has been compressed into three markers, which, quantitatively evaluated, hold a strong prognostic value in stage I-III CRC patients. Fewer studies are available on the metastatic setting, namely CLM, although much inspiration is taken from the evidence collected for primary CRC. One important question is whether primary CRC lesions and their hepatic metastases display comparable immune landscapes. If this was the case, one could capture relevant information on the immune environment of the metastatic nodule by analyzing the primary tumor [49]. Another critical issue is related to the heterogeneity of the nodules, when more than one is surgically resected. Evidence has been collected that heterogeneity is dominant both among primary and paired metastatic tumor and among synchronous metastases [49,50]. This observation is in line with the mixed response observed in same patients after treatment, whereby some lesions regress and other do not. Notwithstanding these issues, several lymphoid-markers have been tested as prognostic, including CD3, CD8, CD4, granzyme B and generally associated to favorable outcome [5,51,52,53], while the impact of T regulatory cell marker FoxP3 is less clear [54,55]. Recently, a study assessed the prognostic value of a consensus Immunoscore, RAS mutational status and pathological score (PS) combining relevant clinicopathological parameters (including R1 positive margins, number of lesions, replacement or mixed growth pattern and steatohepatitis) in a large cohort of 221 CLM patients [2]. Immunoscore remained the major determinant of overall survival by itself, confirming the strength of the adaptive immunity in controlling tumor recurrence, while PS stratified patients for tumor recurrence but was not related to survival. Efforts are focused on enhancing the fitness of clinical predictors and integration of several parameters into relevant scores.

### 3.2. The Potential of Macrophages as Prognostic Factors: Not an Easy Task

The attention reserved to macrophages as critical orchestrators of pro-tumor circuits in liver diseases is being translated into efforts aimed at testing these phagocytes as prognostic factors in colo-rectal liver metastasis [48,56]. Macrophages are important immune regulators in the microenvironment of many tumors [57,58]. Most of the available studies have documented pro-tumor functions. However, a large body of evidence is also available suggesting that they can mediate anticancer strategies and they are currently being evaluated as potential targets of cancer immunotherapy [59]. Collectively, there is strong enthusiasm on the possibility to identify macrophage-based prognostic classifiers, but this task has been encountering several obstacles.

Almost every tumor tissue is populated by macrophages, which can be either the result of local differentiation and maturation processes from circulating monocytic precursors, or they can represent the fraction of macrophages resident in the tissue. This ontogeny-related difference has important implications in terms of prognostic studies. For instance, except for a few situations in which a specific marker is available allowing a clear discrimination of recruited versus resident macrophages (primarily available in murine preclinical models), question is whether it is correct to evaluate both the components. Tissue-resident macrophages of the liver, commonly referred to as Kupffer cells, are traditionally considered macrophages with peculiar tissue tasks [60], thus expected to be quite distinct form recruited macrophages and also tolerogenic and immunosuppressive by nature, while the recruited macrophages could acquire this phenotype upon exposure to microenvironmental stimuli. Both Kupffer cells and mature macrophages have a strong expression of the common macrophage markers, including CD68 and CD163. Operationally, a quantitative assessment of density of macrophages in a CLM tissue performed by computing immunoreactive area of CD68 would not allow to distinguish among resident and recruited macrophages, thus mixing up to types of cells that could potentially be fairly different. Human studies so far have not allowed clarifying the relative contribution of recruited and resident macrophages in the microenvironment of CLM. Despite the fact that recent high-dimensional studies have shed light on the variety of immune cells in human CRC tissues [61], fully elucidating the complex dynamics and relative contribution of resident versus recruited immune components requires further studies.

Paying more attention to localization in the tissue context and the interaction with various types of endothelial cells could be the way forward. Macrophages occupy very specific regions in the liver as well as in tumor tissues, such as the sinusoidal space, the peritumor region, the tumor core, or the invasive margin [62]. Kupffer cells hold a strategic position in the sinusoids, where they can also project towards the perisinusoidal space of Disse [63], and their density decreases approaching the tumor nodule, as the liver tissue becomes replaced by the tumor one. In contrast, monocyte-derived macrophages are more commonly found in the portal triad and their density dramatically increases closed to the tumor region. In an image-based approach, such differences could be taken into account and allow discriminating between various populations. The relationship between macrophages and vessels in the liver environment could be also critical in terms of pro-metastatic capability because a population of VEGFR1+ macrophages has been shown to be pro-angiogenic and correlate with progression of disease in human CLM [64].

### 3.3. Macrophage Morphology: A New Feature to Be Considered?

**Figure 2:**
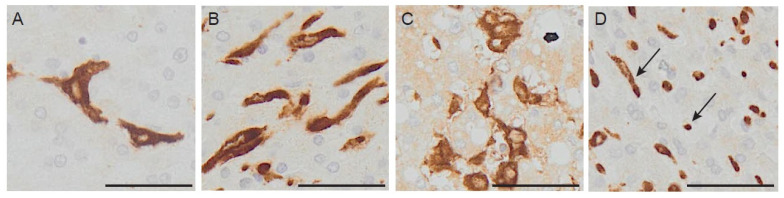
Immune-markers in human colorectal liver metastases. Morphology of macrophages in CLM tissue. Section of human colo-rectal liver metastasis stained with CD163, showing the coexistence of different morphological types in the same region. Panel (**A**) shows spiky macrophages, panel (**B**) shows elongated macrophages, in panel (**C**) there is abundance of large macrophages with intracellular vacuoles and in panel (**D**) both small round macrophages and elongated ones (arrow). Scale bar: 200 μm.

The dramatic advancement of image-based profiling is revolutionizing our approach to prognostication. High-dimensional cell profiling allows capturing multiple features of a cell population, or even of single cells, and finds applicability in high-throughput screenings, drug discovery systems, immuno-monitoring studies [65]. Several high-dimensional readout technologies can be used to build up a feature profile. Image-based approaches are very suitable for the assessment of immune cells in cancer tissues because they allow retaining the important information related to localization of immune cells in the tissue, their interaction with the extracellular matrix and components, the relative position towards other immune mediators. These systems are based on the identification of relevant immune features to be combined in the profiling. Recently, macrophage morphology has gained attention, as an additional parameter capturing distinct macrophage populations with prognostic functions [66]. Immunologists are trained to detect subtle differences in cell morphological features and attribute them to variations in activation states of the cells analyzed, thus using cell morphology as a proxy of their activation. This skill turns very helpful in daily routine, when it allows quickly checking on the activation of cultured cells without disturbing the culture itself. Apparently, cell morphology is linked to function. Is there a causative link? It seems so for macrophages, which modify their shape in vitro, when exposed to polarizing agents [67], or in vivo in fibrotic and chronically inflamed tissues [68,69]. This hypothesis has been tested in a recent study on colo-rectal liver metastasis, in which morphology of macrophages has been systematically quantified and correlated to prognosis. Distinct populations of small and large macrophages were identified (Figure 2), with very different transcriptional profiles [66] and when tested as a prognostic tool, they showed opposite correlation with clinical outcome. Ongoing studies aimed at evaluating macrophage features as prognostic or predictive markers would benefit from digital tools and machine-learning based approaches.

## 4. Conclusions

With the development of new surgical techniques and more effective therapeutic protocols, eligibility of CLM patients for surgical resection has progressively increased over years. While drastically lowering morbidity and mortality rates, this improvement in the management of CLM patients imposes identification of prognostic classifiers, to help clinical oncologists as well surgeons in selecting the best therapeutic options and follow patients’ response. Recent successful studies describing the prognostic and predictive value of immune mediators in primary colo-rectal cancer have fueled the interest in the development of immune-based tools to aid in the critical prognostication process in colo-rectal liver metastasis. Further efforts are needed to understand how much of the complexity of immune cells in the spotlight can be exploited to identify nonredundant markers of disease progression. The value of immune mediators is being evaluated alongside more common histopathological parameters, whose impact on survival of CLM patients is still controversial. Provided that quantifiable parameters are generated, these could be integrated into prognostic scores, potentially complementing the ones available.
